# Scribble Acts in the *Drosophila* Fat-Hippo Pathway to Regulate Warts Activity

**DOI:** 10.1371/journal.pone.0047173

**Published:** 2012-11-05

**Authors:** Shilpi Verghese, Indrayani Waghmare, Hailey Kwon, Katelin Hanes, Madhuri Kango-Singh

**Affiliations:** 1 Department of Biology, University of Dayton, Dayton, Ohio, United States of America; 2 Pre-Medical Programs, University of Dayton, Dayton, Ohio, United States of America; 3 Center for Tissue Regeneration and Engineering at Dayton, University of Dayton, Dayton, Ohio, United States of America; Baylor University, United States of America

## Abstract

Epithelial cells are the major cell-type for all organs in multicellular organisms. In order to achieve correct organ size, epithelial tissues need mechanisms that limit their proliferation, and protect tissues from damage caused by defective epithelial cells. Recently, the Hippo signaling pathway has emerged as a major mechanism that orchestrates epithelial development. Hippo signaling is required for cells to stop proliferation as in the absence of Hippo signaling tissues continue to proliferate and produce overgrown organs or tumors. Studies in *Drosophila* have led the way in providing a framework for how Hippo alters the pattern of gene transcription in target cells, leading to changes in cell proliferation, survival, and other behaviors. Scribble (Scrib) belongs to a class of neoplastic tumor suppressor genes that are required to establish apical-basal cell polarity. The disruption of apical-basal polarity leads to uncontrolled cell proliferation of epithelial cells. The interaction of apical basal polarity genes with the Hippo pathway has been an area of intense investigation. Loss of *scrib* has been known to affect Hippo pathway targets, however, its functions in the Hippo pathway still remain largely unknown. We investigated the interactions of Scrib with the Hippo pathway. We present data suggesting that *Drosophila scrib* acts downstream of the Fat (Ft) receptor, and requires Hippo signaling for its growth regulatory functions. We show that Ft requires Scrib to interact with Expanded (Ex) and Dachs (D), and for regulating Warts (Wts) levels and stability, thus placing Scrib in the Hippo pathway network.

## Introduction

Growth and differentiation need to be precisely controlled during development to generate organs of appropriate size [1]. The Hippo pathway has emerged as a pathway that regulates growth and organ size in *Drosophila* and mammals [2], [3], [4], [5]. The Hippo pathway regulates organ size by controlling the activity of the transcriptional co-activator Yki in flies (and YAP/TAZ in mammals), which is an important regulator of proliferation and apoptosis [2], [3], [4], [5]. The expanding roles of Hippo signaling now include regulation of cell competition, compensatory proliferation, regeneration and stem-cell renewal [2], [3], [4], [5]. Emerging data implicates genes controlling cell polarity, cell adhesion and cell-cell junctions as important components of the Hippo pathway [2], [3], [4], [5].

The Hippo pathway comprises of a core kinase cascade involving the Ste-20 family kinase Hippo [6], [7], [8], [9], and the DMPK family kinase Warts (Wts) [10], [11], which in turn regulates the transcriptional co-activator Yorkie (Yki) [12]. Nuclear availability of Yki is regulated by phosphorylation-dependent and -independent mechanisms [12]. Active Yki translocates to the nucleus, where it forms a complex with the transcription factor Scalloped (Sd) [13], [14], [15] [or Mothers against Dpp (MAD), Teashirt (Tsh) or Homothorax (Hth)] [16] to induce the expression of target genes that promote (a) cell proliferation and cell survival like the *bantam miRNA*, *myc*, (b) cell cycle progression *e.g.*, E2F1, *cyclins A, B, E,* and (c) inhibitors of apoptosis like *drosophila inhibitor of apoptosis (diap1)* [2], [3], [4], [5]. Hippo signaling also regulates the expression of several genes within its pathway like *ex, mer, kibra, crb*, and *fj* via a negative feedback loop [2], [3], [4], [5]. This vast repertoire of target genes confers tremendous versatility to Hippo signaling and also allows context-dependent response of Hippo signaling activity.

Multiple points of signal integration are beginning to emerge in the Hippo pathway. For example, upstream of Hpo multiple apical determinants feed into the Hippo pathway [17], which include Expanded (Ex), Merlin (Mer), Crumbs (Crb), Kibra, and the atypical cadherin Fat (Ft). In addition, the Ras-association family protein (dRASSF); the apico-basal polarity proteins Lethal giant larvae (Lgl) [18], and atypical Protein Kinase C (aPKC) [18]. The immunoglobulin domain-containing cell-adhesion molecule Echinoid (Ed) [19] also acts upstream of Hpo. In addition some components feed into the Hippo pathway at the level of the Wts kinase. These include the atypical myosin Dachs (D) [20] which together with the LIM domain protein Zyxin (Zyx) [21] to regulate Wts levels [17]. Thus, the Hippo signaling cascade responds to multiple and diverse stimuli which comprise a variety of receptor and non-receptor proteins.

Recently, the tumor suppressor gene Scribble (Scrib) was shown to participate in the Hippo signaling pathway [22], [23], [24]. Scrib, along with Lethal giant larvae (Lgl), Discs large (Dlg), belongs to a class of neoplastic tumor suppressor genes that are required to establish apical-basal cell polarity and growth control [25]. The disruption of apical-basal polarity leads to uncontrolled cell proliferation of epithelial cells, and results in an epithelial-to-mesenchymal transition (EMT) that underlies the development of cancer [26], [27], [28]. Lgl, Dlg, and Scrib, are adaptor proteins each with multiple protein-protein interaction motifs such as PDZ domains and they localize to the basolateral membrane basal to adherens junctions [29], [30], [31], [32], [33]. Dlg binds to Scrib [34] and all three are required for the proper organization and localization of other apical-basal polarity genes. Recent studies indicate that the neoplastic tumor suppressor genes directly regulate cell proliferation of epithelial cells, rather than indirectly through effects on the localization of growth factor receptors [35], [36], [37], because hypomorphic conditions for Lgl and Dlg, for example, affect growth without affecting cell polarity [38], [39].

Using morpholinos in zebrafish embryos, and human and mammalian tissue culture cells it was shown that Scrib physically interacts with mammalian Fat1 and *Drosophila* Fat, and inhibits YAP1-dependent luciferase expression as effectively as Lats2 [23]. *Drosophila* Hippo signaling pathway is implicated in the differentiation and polarity of the follicular epithelia during oogenesis, where components of the basolateral junctions (*scrib*) signal to the downstream Warts kinase [22]. In vertebrates, four Fat genes partition different signaling functions and Scribble seems to promote both PCP and Hippo signaling pathways [40], [41].

However, the molecular pathway through which the neoplastic tumor suppressor genes control cell proliferation *in-vivo* remains unclear. We present data suggesting that *Drosophila scrib* acts downstream of the Fat receptor, and requires Hippo signaling for its growth regulatory functions. We show that Ft requires Scrib to interact with Ex and Dachs, and for regulating Wts levels and stability, thus placing Scrib in the Hippo pathway network.

## Materials and Methods

### Ethics Statement

No specific permits were required for the described field studies.

### Fly Stocks

All stocks used in this study have been described earlier. We used the following fly lines: yw; FRT82B scrib^2^/TM6B, yw; FRT82B scrib^J7B3^/TM6B, w; scrib^3^/TM6B, w; scrib^7^/TM6B, yw; ft^fd^ FRT40A/CyO, yw; ft^422^ FRT40A/CyO, yw; ex^697^/CyO, yw; ex^BQ^ FRT40A/CyO, yw; FRT42D yki^B5^/CyO, eyFlp; Act>y+>GAL4 UASGFP; FRT82B Tub-GAL80, yw hsFlp; FRT82B wts^X1^/TM6B, yw; FRT82B scrib^2^ wts^X1^/TM6B, eyFlp; FRT82B, yw hsFlp; FRT82B ubiGFP/TM6B, UbxFlp; ubiGFP FRT40A/CyO, UAS-Scrib^RNAi^ (Bloomington # 29552), UAS-D (Bloomington # 28814), UAS-Ft^RNAi^ (VDRC # V9396) [42], UAS-Sd^RNAi^ (Bloomington # 29352), UAS-Yki-V5 (Bloomington # 28819), UAS-GFP and nub-Gal4. All crosses were performed at 25°C unless otherwise mentioned. All RNAi lines were tested for specific effects on their corresponding genes by testing for downregulation of gene expression using specific antibodies, or by genetic interactions/rescue experiments with known mutants. Adult flies were photographed using the Zeiss apotome microscope and Axivision software.

### Immunohistochemistry

The eye and wing imaginal discs were dissected in PBS, fixed in 4% paraformaldehyde for 20 min at room temperature, washed in PBST (PBS + 0.2% TritonX-100) 2X10min, blocked using normal goat serum, and incubated with primary antibodies overnight at 4°C. The following primary antibodies were used mouse-anti DIAP1 (1∶250); mouse-anti βgal (1∶100), rabbit-anti Ex (1∶1500), guinea pig-anti Scrib (1∶500).The following secondary antibodies (Jackson Immunoresearch) were used: anti-mouse Cy3 (1∶1000), anti-rabbit Cy3 (1∶1000) and anti-guinea pig Cy3 (1∶1000).

### Western Blotting

Wing imaginal discs were dissected in cold PBS, and lysed on SDS sample buffer (Lanes 1–4). For the sample in Lane 5 whole larva extracts were prepared from double mutant larvae in order to compare data. Western blots were performed according to standard protocols. The antibodies used were rb- anti Wts (1∶1500) and m- anti-α Tubulin (Sigma, 1∶1000). ECL reactions were developed using the GE Healthcare ECL Kit according to manufacturer’s instructions, and images were captured using the Bio-Spectrum^(R)^ 500 Imaging System or HyBlot CL auto-radiaography film.

### Adult Wing Mounting and Imaging

Adult flies were collected in 70% Ethanol and dehydrated in an ascending alcohol series. Completely dehydrated flies were used for wing processing. The clipped wings were mounted in Canada Balsam (3∶1 Canada Balsam: Methyl Salicylate). The wing images were taken using Olympus BX51 Microscope mounted with an Olympus XM10 camera and CellSens Dimensions Software.

## Results

### *Scrib* Genetically Interacts with the Hippo Pathway

**Figure 1:**
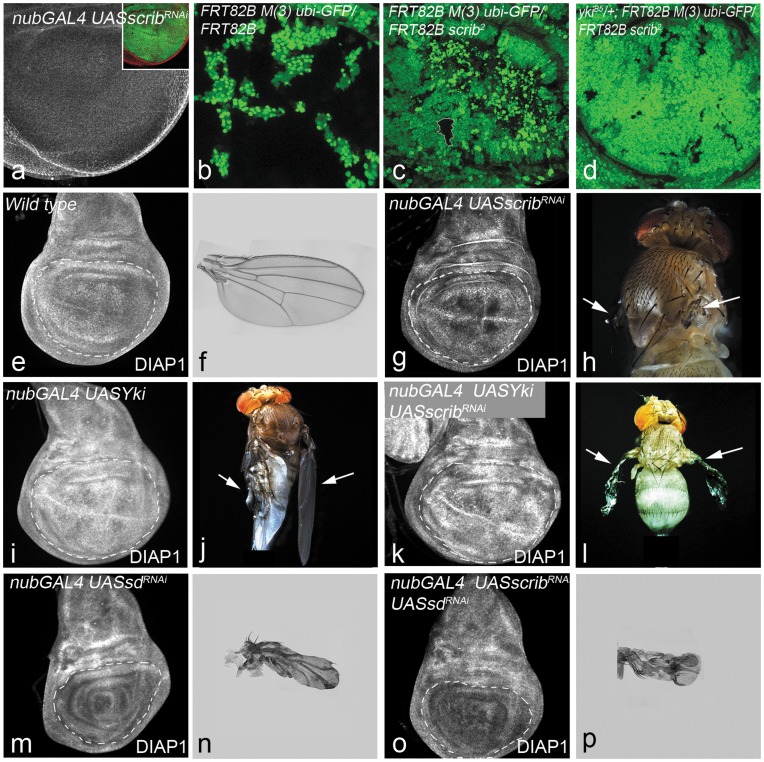
*scrib* interacts with *yki* to regulate growth. (**a**) *nub-GAL4 UAS-scrib^RNAi^ UAS-GFP* wing imaginal discs showing down-regulation of Scrib expression in the *nub-Gal4* domain (shown by GFP expression in the inset). (b–d) Panels show comparison of clone sizes of GFP negative clones from larvae of the following genotype: *ubx-Flp; FRT82B M(3)95A ubi-GFP/FRT82B* (b), *ubx-Flp; FRT82B M(3)95A ubi-GFP/FRT82B scrib^2^* (c), and *ubx-Flp; yki^B5^/+; FRT82B M(3)95A ubi-GFP/FRT82B scrib^2^* (d). Diap1 expression in third instar wing imaginal disc from wild-type (e), *nub-GAL4 UAS-scrib^RNAi^* (g), *nub-GAL4 UAS-Yki* (i), *nub-GAL4 UAS-Yki UAS-scrib^RNAi^* (k) *nub-GAL4 UAS-sd^RNAi^* (m), *nub-GAL4 UAS-scrib^RNAi^ UAS-sd^RNAi^* (o) larvae. The corresponding adult phenotypes for all genotypes are shown in panels to the right of imaginal discs. Adult wings of wild-type (f), *nub-GAL4 UAS-sd^RNAi^* (n), and *nub-GAL4 UAS-scrib^RNAi^ UAS-sd^RNAi^* (p) are shown. Images of adult flies are shown for *nub-GAL4 UAS-scrib^RNAi^* (h), *nub-GAL4 UAS-Yki* (j), *nub-GAL4 UAS-Yki UAS-scrib^RNAi^* (l).

*scrib* mutant clones are small and tend to be eliminated by cell competition in flies [24], [43], [44], [45], [46], [47] and in mammalian epithelial cells [48]. Therefore, we used a *UAS-scrib^RNAi^* transgene to effectively generate large *scrib* mutant patches (Fig. 1a) that can be evaluated for effects of loss of *scrib* alone [18], [24]. We wanted to use this approach to assay the effects of loss of *scrib* alone, unlike other studies where the clone size of *scrib* mutant cells was enhanced by preventing cell death by over-expressing p35- a pan caspase inhibitor [43], [45] or Bsk^DN –^ the *Drosophila* Jun- Kinase [43], [44], [46], [47] or by making *scrib* mutant clones in an *eiger* (the *Drosophila* TNF superfamily ligand) mutant background [45], [46], [47]. Using the wing pouch specific *nub-Gal4* driver, we over-expressed *UAS-scrib^RNAi^* in the developing wing imaginal discs (Fig. 1a). Knocking down *scrib* levels (Fig. 1a) results in reduction of the wing pouch size (Fig. 1g) in the imaginal discs, and the development of adult flies with rudimentary wings (Fig. 1h, arrows).

Next we tested for genetic interactions of *scrib* with members of the Hippo signaling pathway. Scrib requires *yki* to regulate cell proliferation as the growth of *scrib* mutant discs is strongly suppressed by heterozygosity of *yki* [24]. Loss of function clones of *scrib* also shows a similar requirement for *yki* function (Fig. 1b–d), as the size of the *scrib* mutant clones (Fig. 1c) is dominantly suppressed by heterozygosity of *yki* (Fig. 1d). We next tested the Yki and Scrib interaction by over-expressing Yki (*UAS-Yki*) in *nub-Gal4 UAS-scrib^RNAi^* wing imaginal discs (Fig. 1i–l). *nub-Gal4 UAS-scrib^RNAi^ UAS-Yki* wing discs (Fig. 1k) showed overgrown wing pouch similar to those of discs overexpressing *UAS-Yki* alone (Fig. 1i). Animals of *nub-Gal4 UAS-Yki* (Fig. 1j) and *nub-Gal4 UAS-scrib^RNAi^ UAS-Yki* (Fig. 1l) genotypes developed to pharates with large crumpled wings. Thus consistent with earlier observations, changes in Yki levels affect *scrib* phenotypes both in terms of clone size and cell survival [24], [44], [45], [46], [47]. We next tested if *scrib* interacts with *sd*, the transcription factor that binds with Yki. Loss of *sd* expression (*UAS-sd^RNAi^*) causes reduction in the wing pouch in the imaginal discs (Fig. 1m) and formation of very small stubby-wings in the adults (Fig. 1n). Wing imaginal discs where both *scrib* and *sd* functions were knocked down (*nub-Gal4 UAS-scrib^RNAi^ UAS-sd^RNAi^)* had a small pouch (Fig. 1o) similar to those of discs over-expressing *UAS-sd^RNAi^* alone (Fig.1m), and showed the stubby-wing phenotype in the adult (Fig. 1p).

To further assess the interactions of *scrib* with Hippo pathway, we tested the levels of expression of DIAP1, a member of the intrinsic cell death pathway, and a transcriptional target of the Hippo pathway [2], [3], [4], [5]. DIAP1 protein is induced in cells where Hippo signaling is down-regulated [2], [3], [4], [5]. Over-expression of *UAS-scrib^RNA^* ^i^ causes downregulation of DIAP1 (Fig. 1g), which possibly contributes to the elimination of *scrib* mutant cells by cell competition. Over-expression of Yki alone (Fig. 1i) or co-expression of Yki with *UAS-scrib^RNAi^* (Fig. 1k) leads to robust induction of DIAP1, suggesting that *yki* acts epistatically to *scrib*. Downregulation of *sd* alone (Fig. 1m) or together with *scrib* (Fig. 1o) leads to downregulation of DIAP1 in imaginal discs, suggesting that *sd* genetically acts downstream of *scrib.* Taken together, these data showed that Yki and Sd act downstream of Scrib, and are required for its growth regulatory functions.

**Figure 2:**
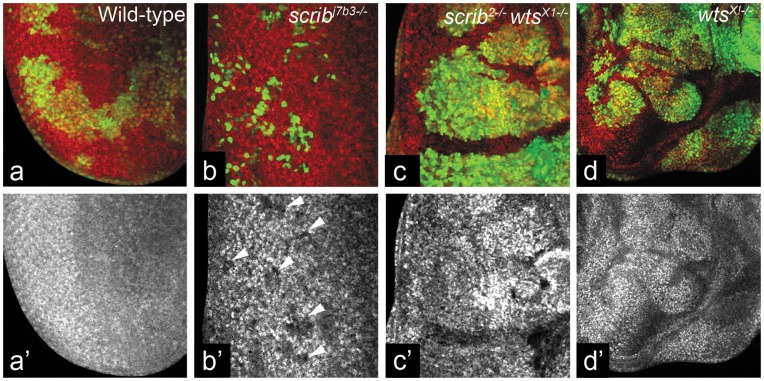
*scrib* acts upstream of *wts*. (a–d) Panels show comparison of MARCM clones (GFP-positive) from wild-type (a), *scrib* mutant (b), *scrib wts* double mutant (c), and *wts* mutant (d) eye imaginal discs. DIAP1 expression is shown in red in a–d and in greyscale in a’–d’. Anterior is to the right, and magnification is same in all images. Genotypes: (a) *ey Flp; Act>y+>GAL4 UAS-GFP; FRT82B tub-GAL80/FRT82B* (b) *ey Flp; Act>y+>GAL4 UAS-GFP; FRT82B tub-GAL80/FRT82B scrib^j7b3^* (c) *ey Flp; Act>y+>GAL4 UAS-GFP; FRT82B tub-GAL80/FRT82B scrib^2^ wts^X1^* (d) *ey Flp; Act>y+>GAL4 UAS-GFP; FRT82B tub-GAL80/FRT82B wts^X1^.*

We then tested if *scrib* interacts with other Hippo pathway components that act upstream of Yki. We first tested *scrib wts* interaction by comparing the clone size of *wts* mutant clones, to *scrib wts* double mutant clones. The *scrib* mutant clones (Fig. 2b) are slow growing and small, whereas the *wts* mutant clones show dramatic overgrowth (Fig. 2d). The *scrib wts* double mutant clones also show robust overgrowths (Fig. 2c). Next, we tested the expression of DIAP1 levels in *scrib wts* double mutant clones. DIAP1 is suppressed in *scrib* mutant cells (Fig. 2b’, arrowheads) and induced strongly in *wts* mutant cells (Fig. 2d’). Interestingly, the *scrib wts* double mutant clones induce DIAP1 expression (Fig. 2c’). Based on clone size and the regulation of DIAP1 expression, *wts* acts downstream of *scrib* as the *scrib wts* double mutant clones show phenotypes similar to loss of *wts* alone. The genetic interactions so far suggested that *scrib* maybe another input upstream of Wts in the Hippo pathway. Therefore, we (1) tested the effects of loss of *scrib* on the transcriptional targets of Hippo pathway, and (2) tested genetic epistasis interactions between upstream Hippo components and *scrib*.

### *Scrib* Mutant Cells Upregulate Hippo Pathway Target Genes

**Figure 3:**
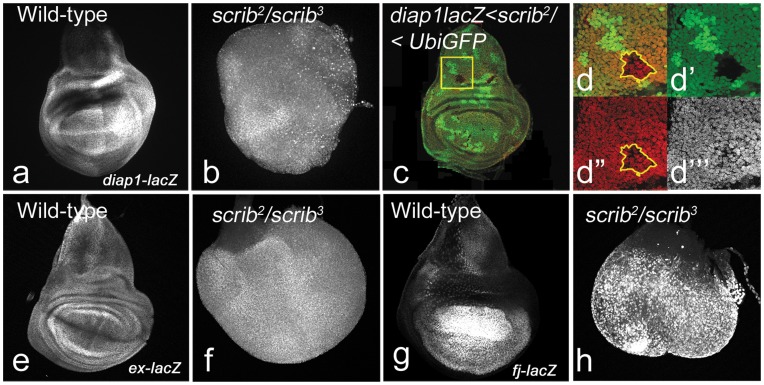
Hippo target genes are affected in *scrib* mutant cells. (a, e, g) Panels show *diap1-lacZ* (a), *ex-lacZ* (e), *fj-lacZ* (g) expression in wild-type wing imaginal discs. (b, f, h) *scrib^2^/scrib^3^* mutant homozygous discs showing *diap1-lacZ* (b), *ex-lacZ* (f), *fj-lacZ* (h) expression. (c–d’’’) *diap-lacZ* levels in scrib mutant clones in wing imaginal discs from *yw hs-Flp FRT82B scrib^2/^FRT82B ubi-GFP* larvae (c). Note that a majority of the clones get eliminated 48h after induction in the wing pouch. (d–d’’’) Magnified view of the clone in the notum indicated by a yellow box. The clone (GFP negative) is smaller than its wild-type (2XGFP) twin-spot, and shows down-regulation of *diap-lacZ* (red in d’’ and greyscale in d’’’). The clone boundary is marked by yellow line (d,d”). The magnification and orientation of images in a–c, e–h is identical.

*scrib* loss of function clones are slow-growing [24], [43] and are competed out by the surrounding wild-type cells [49]. We compared the effects of complete loss of *scrib* in imaginal discs (Fig. 3b,f,h) to loss of *scrib* in mutant clones generated by the FLP/FRT system in small patches in the eye (data not shown) and wing disc (Fig. 3d–d’’’). Loss of *scrib* throughout the wing imaginal discs in homozygous mutant larvae leads to neoplastic overgrowths during the extended larval life (Fig. 3b,f,h). Consistent with earlier reports, *scrib* mutant clones (Fig. 3c,d) are small compared to the wild-type twin clones (Fig. 3d’’), and are sparsely represented due to their elimination by the neighboring wild-type cells (Fig. 3c) [24], [43], [49]. We tested levels of expression of transcriptional targets of Hippo signaling (*viz., diap1-lacZ, ex-lacZ, fj-lacZ*) in homozygous *scrib* mutant discs and in *scrib* mutant clones. In wild-type, the expression of *diap1-lacZ* (Fig. 3a) and *ex-lacZ* (Fig. 3e) is ubiquitous throughout the wing imaginal disc, and *fj-lacZ* shows a gradient in the wing pouch with the strongest expression at the wing margin and diminishing levels of expression from the margin to the wing hinge (Fig. 3g). In *scrib* homozygous mutant discs the expression of *diap1-lacZ* (Fig. 3b), *ex-lacZ* (Fig. 3f), and *fj-lacZ* (Fig. 3h) is upregulated. A majority of *scrib* clones are eliminated, and expression levels of Hippo target genes is not affected in small clones (∼2–6 cells per clone) (data not shown). However, in larger clones (∼15 cells per clone) the expression of *diap1-lacZ* (Fig. 3d,d’) is down-regulated in the mutant cells and induced non-cell autonomously around the mutant clone. This is consistent with earlier observations that Hippo activity is not uniformly upregulated in *scrib* mutant cells facing cell competition [24], [45]. These data suggest that loss of *scrib* in homozygous mutant discs and in mutant clones affects expression of Hippo target genes, and Hippo pathway activity.

### Scrib Acts Downstream of Fat in the Hippo Pathway

**Figure 4:**
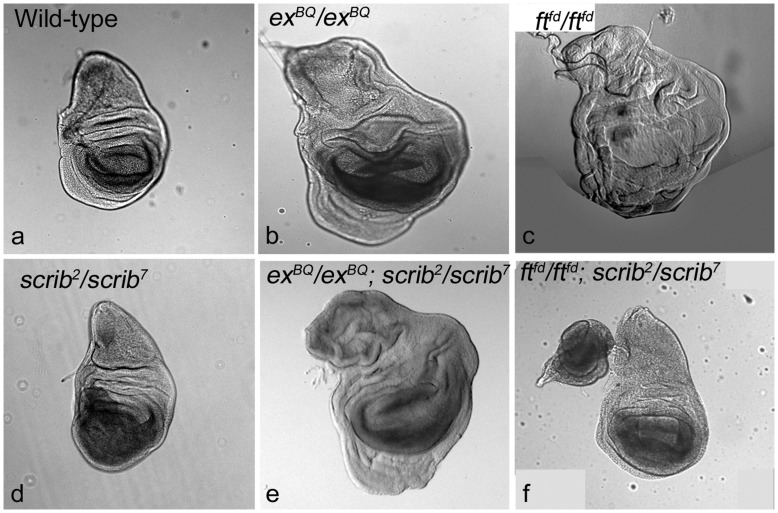
*ft* requires *scrib* to regulate growth. Wing imaginal discs from (a) wild-type, and homozygous mutant larvae of (b) *ex^BQ^ FRT40A^/^ex^BQ^ FRT40A,* (c) *ft^fd^ FRT40A*/*ft^fd^ FRT40A*, and (d) *FRT82B scrib^2^*/*scrib^7^* genotypes are shown. (e,f) Panels show the wing imaginal from double mutant larvae of (e) *ex^BQ^ FRT40A*/*ex^BQ^ FRT40A*; *FRT82B scrib^2^*/*scrib^7^* and (f) *ft^fd^ FRT40A*/*ft^fd^ FRT40A*; *FRT82B scrib^2^*/*scrib^7^* genotypes. Note that the overgrowth induced by loss of *ft* (c) is suppressed by concomitant loss of *scrib* (f). The magnification and orientation of images is identical.

Next, we tested if *scrib* acted within the Hippo pathway (Fig. 4); we generated double mutant combinations of *ex, ft,* and *scrib*. *scrib, ex* and *ft* mutant larvae enter a phase of extended larval development and do not pupate. Compared to wild type (Fig. 4a), transallelic combinations of null and hypomorphic *scrib* mutant alleles (*e,g., scrib^2^/scrib^7^*) leads to development of neoplastic growth in the wing pouch of imaginal discs (Fig. 4d), whereas loss of *ex* (Fig. 4b) or *ft* (Fig. 4c) cause massive hyperplasia. *ex* acts upstream of Hpo and interacts with Yki via multiple mechanisms [3], [17]. We found that the *ex; scrib* double mutants showed *ex*-like hyperplastic phenotypes (Fig. 4e) suggesting that *scrib* may act upstream or parallel to *ex*. Interestingly, *ft scrib* double mutants showed dramatic reduction of overgrowth (Fig. 4f) compared to loss of *ft* alone (Fig. 4c) and resemble *scrib* mutant wing imaginal discs (Fig, 4d). This suggests that *scrib* acts downstream of *ft,* and *ft* may require *scrib* to interact with Ex or Dachs to regulate Yki.

**Figure 5:**
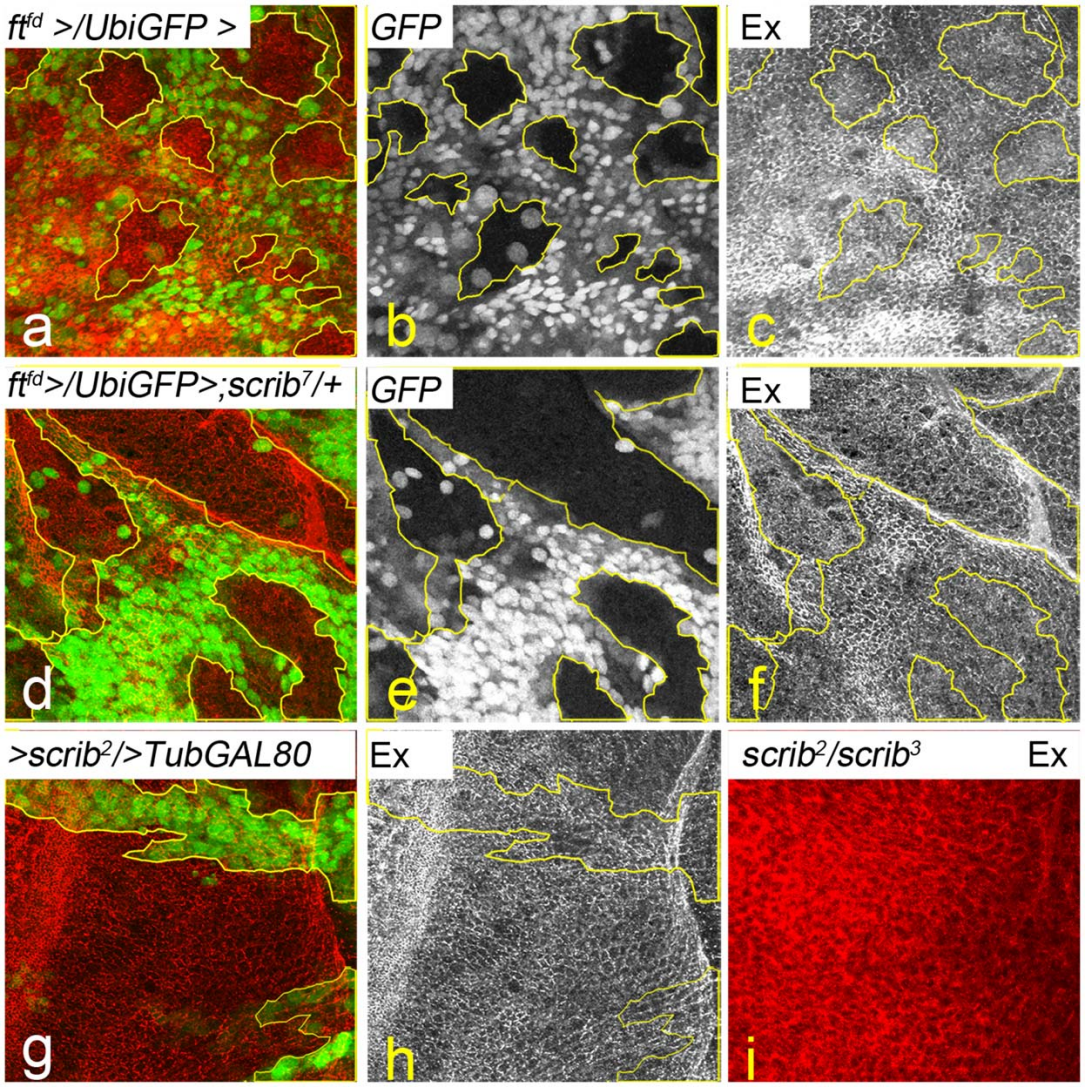
Effect of Ft-Scrib interaction on Ex localization. Ex expression in **(**a–c) *ft* mutant clones (GFP negative) and (d–f) in *ft* mutant clones (GFP negative) induced in *scrib* heterozygous condition is shown. (g,h) Panels show Ex levels in *scrib* mutant clones (g, GFP positive) induced using the MARCM system, and in (i) *scrib* homozygous mutant discs. Ex levels are shown in red (a,d,g,i) and in grey scale (c,f,h). All images are at identical magnification. Genotypes: (a–c) *ubx-Flp; ft^fd^ FRT40A/ubi-GFP FRT40A* (d–f) *ubx-Flp; ft^fd^ FRT40A/ubi-GFP FRT40A; scrib^7^red e/+* (g,h) *ey-Flp; Act>y+>GAL4 UAS-GFP; FRT82B scrib^2^/FRT82B TubGal80* (i) *scrib^2^/scrib^3^.*

We next tested if the *ft scrib* interaction affects the Ft-Ex interaction. Earlier studies have shown that Ex is mislocalized from the apical membrane in *ft* mutant cells (Fig. 5a–c) suggesting that *ft* affects the stability and localization of Ex at the plasma membrane [21], [50], [51], [52], [53]. Loss of *scrib* in mutant clones does not cause loss of apical-basal polarity (Fig. 5g) and Ex is not mislocalized from the membrane (Fig. 5h). However, Ex is mislocalized from the apical membrane and appears cytoplasmic in imaginal discs from homozygous *scrib* mutant animals (*scrib^2^/scrib^3^*) that show neoplastic overgrowth and loss of apical basal polarity (Fig. 5i). To test if Scrib affects signaling from Ft to Ex, we generated *ft* mutant clones in *scrib* heterozygous background (Fig. 5d–f). Loss of *ft* in *scrib* heterozygous background (Fig. 5d, e) does not affect levels or localization of Ex (Fig. 5f) within *ft* mutant clones. Together, these data suggest that Scrib does not affect the Ft-Ex interaction.

### Scrib Acts in the Fat Branch of the Hippo Pathway

**Figure 6:**
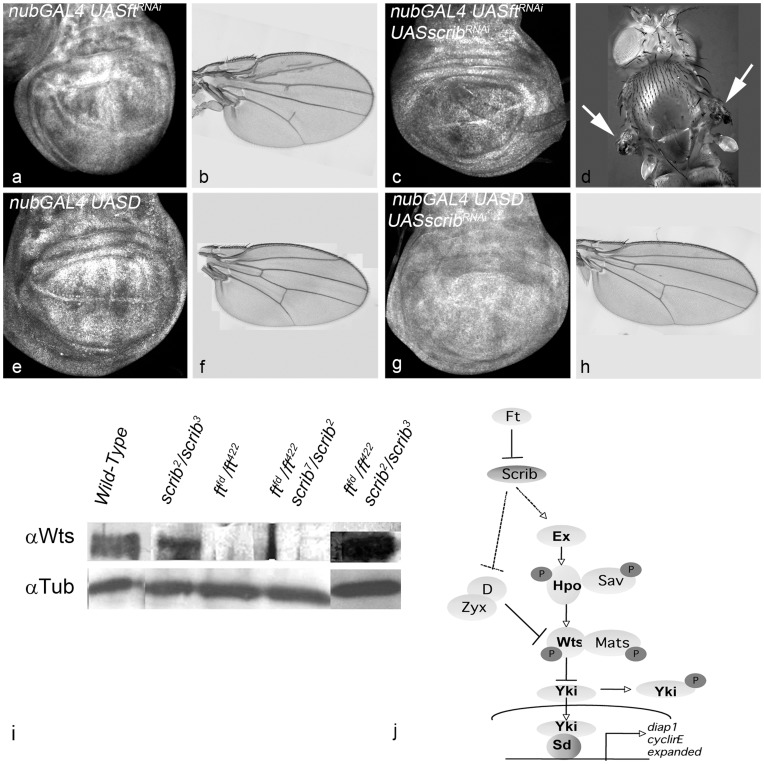
*scrib* interacts upstream of *d* in the Fat-Hippo pathway. Panels show wing imaginal discs from *nub-GAL4 UAS-ft^RNAi^* (a), and *nub-GAL4 UAS-ft^RNAi^ UAS-scrib^RNAi^* (c), *nub-GAL4 UAS-D* (e) and *nub-GAL4 UAS-D UAS-scrib^RNAi^* (g) larvae stained for DIAP1. Adult wings of the corresponding genotypes are shown in (b) *nub-GAL4 UAS-ft^RNAi^*, (f) *nub-GAL4 UAS-D,* and (h) *nub-GAL4 UAS-D UAS-scrib^RNAi^*. (d) Phenotype of *nub-GAL4 UAS-ft^RNAi^ UAS-scrib^RNAi^* adults shows loss of wings (white arrows). (i) Semi-quantitative Western blot for Wts levels in wild-type[Lane 1], *scrib* mutants (*scrib^2^/scrib^3^*) [Lane 2], *ft* mutants (*ft^fd^/ft^422^)*[Lane 3], and *ft; scrib* double mutant (*ft^fd^/ft^422^*; *scrib^2^/scrib^7^*)[Lane 4] and (*ft^fd^/ft^422^*; *scrib^2^/scrib^3^*)[Lane 5] is shown. Anti-α-Tubulin is the loading control. (j): Model of *scrib* in Hippo Pathway- *scrib* acts downstream of *ft* and mediates it effects on growth. Magnification of images in panels a-h is identical.

Next, we tested epistatic interactions between *scrib* and genes downstream of *ft* in the Hippo pathway to further characterize the *ft scrib* interaction. We monitored wing size in the adult flies and DIAP1 expression in the *nub-Gal4* domain of the wing imaginal discs from double mutants to analyze the epistatic interactions (Fig. 6). Down-regulation of *ft* leads to upregulation of DIAP1 expression (Fig. 6a) and formation of overgrown adult wings (Fig. 6b), whereas down-regulation of *scrib* leads to reduction in DIAP1 levels (Fig. 1d). In comparison, *ft scrib* double mutant cells show down-regulation of DIAP1 levels (Fig. 6c), and development of flies with rudimentary wings (Fig, 6d). These observations also support a model where *scrib* acts downstream of *ft*. Several genes act downstream of *ft* in the Hippo pathway to regulate the activity of the Wts kinase. *dachs* is required for normal wing and leg growth and acts genetically downstream of *fat* but upstream of *wts* [53], [54], [55]. *dachs* mutations suppress the effects of *fat* mutations on gene expression, cell affinity and growth in imaginal discs. Dachs is suggested as the molecular link between Ft and the core kinase cascade of the Hippo pathway, as it physically associates with Wts in S2 cell lysates [53]. Overexpression of UAS-D (*nub-Gal4 UAS-D*) leads to overgrowth of the wing pouch (Fig. 6e) and formation of overgrown wings in the adult (Fig. 6f). Over-expression of Dachs in *nub-Gal4 UAS-scrib^RNAi^* wing imaginal discs results in overgrowth (Fig. 6g) resulting formation of larger wings in adult flies (Fig. 6h). DIAP1 expression is upregulated in *nub-Gal4 UAS-D* wing discs (Fig. 6e), and in the double mutant discs (Fig. 6g). Based on regulation of DIAP1 expression in wing discs, and the size of adult wings we conclude that *scrib* acts upstream of Dachs.

Taken together, these epistasis interactions show that *scrib* acts in the Fat branch, and that *ft* requires *scrib* to signal to Ex as well as Dachs. We next asked if Scrib affects the ability of Fat to signal to Wts.

### Scrib Negatively Regulates Wts Activity

Fat is known to affect Wts levels by a post-transcriptional mechanism where compared to wild-type, Wts levels are down-regulated in *ft* mutant discs (Fig. 6i) [21], [53]. Thus we tested if loss of *scrib* affects the regulation of Wts levels by the Fat-Hippo pathway. Using semi-quantitative western blots, we tested Wts levels in wild-type, *ft^−/−^, scrib^−/−^* and *ft^−/−^ scrib^−/−^* double mutants (Fig. 6i). Wts levels are downregulated in *ft* mutant (Fig. 6i), compared to wild-type or *scrib^−/−^ or ft^−/−^ scrib^−/−^* double mutants proteins (Fig. 6i).

In summary our results suggest that *scrib* acts in the Fat branch of the Hippo pathway downstream of Ft. This suggests that Ft requires *scrib* to regulate gene expression and growth of imaginal discs.

## Discussion

Apical basal polarity genes have been studied for regulation of cell junctions and growth [5], [25]. It is clear that the regulation of growth is intimately linked to the formation of normal cell junctions and proper cytoskeletal architecture [4], [27], [56]. The Hippo signaling pathway is known for its roles in the regulation of cell proliferation, apoptosis, and in the organization of cytoskeletal architecture [4], [56]. The interaction of apical basal polarity genes with the Hippo pathway has been an area of intense investigation and new links are beginning to emerge between these genes and the regulation of Hippo Pathway [17], [18], [24], [57], [58], [59], [60], [61]. Crumbs, a gene that regulates the apical complex is the most well characterized gene amongst the other apical basal polarity gene in the Hippo Pathway [57], [59], [61], [62], [63]. Lgl and aPKC have also been shown to interact with Hippo Pathway via independent mechanisms [18], [59], [64], [65], [66], [67].

In mammalian systems, TAZ forms a complex with the cell-polarity determinant Scrib, and loss of Scrib or induction of epithelial-mesenchymal transition (EMT), disrupts the inhibitory association of TAZ with the core Hippo kinases MST and LATS [68], [69], [70]. *scrib* (a member of the basolateral protein complex) has been shown to act downstream of Fat4 and is required for zebrafish pronephros development [23]. Interestingly, in fly tissues, the overgrowth of *scrib* mutant clones is dependent on Yki [18], [24]; yet, the molecular mechanisms of this genetic interaction remain unknown.

Here we provide several lines of evidences to show that *scrib* acts within the Hippo signaling and acts downstream of Fat to regulate Wts levels. Several studies have shown that the regulation of the transcriptional co-activator Yki is central to the regulation of Hippo Pathway [2], [3], [4]. Multiple mechanisms of Yki regulation have been shown [12], [71], [72], [73], [74]. The inhibition of Yki by the Wts kinase is postulated to inhibit the ability of Yki to regulate gene expression by preventing its entry into the nucleus [75]. Wts in turn is positively regulated by the Hpo-Sav complex, and negatively regulated by the Zyxin complex [3], [4]. Using regulation of Hippo target genes and size of adult wings as phenotypic assays, we show that *scrib* genetically interacts with Hippo pathway genes and acts upstream of Yki, Wts, Ex and Dachs. In addition, loss of *scrib* affects Yki activity, as transcriptional targets of Hippo pathway are down-regulated in *scrib* mutant cells. Scrib is known to require Yki for its effects on growth regulation in homozygotes [24] and for the growth of *scrib* mutant cells (this study). Thus, overall our studies place *scrib* downstream of Ft within the Fat-Hippo pathway.

Previous studies have shown that Ft is required for the localization of Ex to the plasma membrane and acts genetically and biochemically upstream of Ex, Hpo, Wts, and Yki [50], [51], [52], [53]. Using genetic epistasis interactions we show that *scrib* acts in the Fat branch of the Hippo pathway, downstream of *ft* (Fig. 4, 5, 6). Our data revealed that *ft* requires *scrib* for the regulation of growth (Fig. 4f). Genetic data suggest that Ft also regulates Warts activity through Ex independent pathways, and has implicated the myosin-like molecule Dachs in growth regulation [14], [41], [42]. Dachs acts upstream of Ex and Wts for the regulation of disc growth [53]. Since Ft signaling works via Ex or Dachs, we next checked if *scrib* is required for one or both of these interactions. Our data from genetic epistasis experiments shows that *scrib* acts upstream of D and Ex in the Hippo pathway (Fig. 4, 6). Using the localization of Ex as a criteria, we tested if the mislocalization of Ex in *ft* mutant cells is affected by heterozygosity of *scrib*. We found that heterozygosity of *scrib* leads to no change in the growth of *ft* mutant clones, and Ex localization is not affected. These data suggest that *scrib* genetically acts downstream of Fat and upstream of Dachs in the Fat-Hippo pathway to regulate growth.

Fat regulates Warts protein levels, most likely via Dachs, which can bind and stabilize Warts [20], [53], [55]. Stability of Warts is also affected by the kinase Discs Overgrown (Dco) [53], [76]. Dachs is known to bind and promote the stability of Wts and Zyxin in a protein complex [21]. We tested if *scrib* affects Wts levels and found that loss of *scrib* leads to accumulation of Wts, whereas loss of *scrib* and *ft* together affects the stability of Wts similar to the loss of *ft* (Fig. 6), suggesting that *scrib* is genetically required for the mechanisms through which Ft regulates Wts levels and stability. In conclusion, our studies place *scrib* downstream of *ft* within the Hippo pathway. Given the complex relationship between *ft* and *ex* in the regulation of Wts and Hippo pathway activity [53], [77], [78], in the future it will be interesting to find how signals downstream of Fat are relayed and controlled for regulating pathway activity.

## References

[1] Conlon I, Raff M (1999) Size control in animal development. Cell 96: 235–244. 9988218 10.1016/s0092-8674(00)80563-2

[2] Kango-Singh M, Singh A (2009) Regulation of organ size: insights from the Drosophila Hippo signaling pathway. Dev Dyn 238: 1627–1637. 19517570 10.1002/dvdy.21996

[3] Halder G, Johnson RL (2011) Hippo signaling: growth control and beyond. Development 138: 9–22. 21138973 10.1242/dev.045500 PMC2998162

[4] Staley BK, Irvine KD (2012) Hippo signaling in Drosophila: recent advances and insights. Dev Dyn 241: 3–15. 22174083 10.1002/dvdy.22723 PMC3426292

[5] Boggiano JC, Fehon RG (2012) Growth control by committee: intercellular junctions, cell polarity, and the cytoskeleton regulate Hippo signaling. Dev Cell 22: 695–702. 22516196 10.1016/j.devcel.2012.03.013 PMC3376383

[6] Harvey KF, Pfleger CM, Hariharan IK (2003) The Drosophila Mst ortholog, hippo, restricts growth and cell proliferation and promotes apoptosis. Cell 114: 457–467. 12941274 10.1016/s0092-8674(03)00557-9

[7] Udan RS, Kango-Singh M, Nolo R, Tao C, Halder G (2003) Hippo promotes proliferation arrest and apoptosis in the Salvador/Warts pathway. Nat Cell Biol. 10.1038/ncb1050 14502294

[8] Jia J, Zhang W, Wang B, Trinko R, Jiang J (2003) The Drosophila Ste20 family kinase dMST functions as a tumor suppressor by restricting cell proliferation and promoting apoptosis. Genes Dev 17: 2514–2519. 14561774 10.1101/gad.1134003 PMC218145

[9] Wu S, Huang J, Dong J, Pan D (2003) hippo encodes a Ste-20 family protein kinase that restricts cell proliferation and promotes apoptosis in conjunction with salvador and warts. Cell 114: 445–456. 12941273 10.1016/s0092-8674(03)00549-x

[10] Justice RW, Zilian O, Woods DF, Noll M, Bryant PJ (1995) The Drosophila tumor suppressor gene warts encodes a homolog of human myotonic dystrophy kinase and is required for the control of cell shape and proliferation. Genes Dev 9: 534–546. 7698644 10.1101/gad.9.5.534

[11] Xu T, Wang W, Zhang S, Stewart RA, Yu W (1995) Identifying tumour suppressors in genetic mosaics: the Drosophila lats gene encodes a putative protein kinase. Development 121: 1053–1063. 7743921 10.1242/dev.121.4.1053

[12] Oh H, Irvine KD (2010) Yorkie: the final destination of Hippo signaling. Trends Cell Biol 20: 410–417. 20452772 10.1016/j.tcb.2010.04.005 PMC2919348

[13] Goulev Y, Fauny JD, Gonzalez-Marti B, Flagiello D, Silber J, et al (2008) SCALLOPED interacts with YORKIE, the nuclear effector of the hippo tumor-suppressor pathway in Drosophila. Curr Biol 18: 435–441. 18313299 10.1016/j.cub.2008.02.034

[14] Wu S, Liu Y, Zheng Y, Dong J, Pan D (2008) The TEAD/TEF family protein Scalloped mediates transcriptional output of the Hippo growth-regulatory pathway. Dev Cell 14: 388–398. 18258486 10.1016/j.devcel.2008.01.007

[15] Zhang L, Ren F, Zhang Q, Chen Y, Wang B, et al (2008) The TEAD/TEF family of transcription factor Scalloped mediates Hippo signaling in organ size control. Dev Cell 14: 377–387. 18258485 10.1016/j.devcel.2008.01.006 PMC2292673

[16] Peng HW, Slattery M, Mann RS (2009) Transcription factor choice in the Hippo signaling pathway: homothorax and yorkie regulation of the microRNA bantam in the progenitor domain of the Drosophila eye imaginal disc. Genes Dev 23: 2307–2319. 19762509 10.1101/gad.1820009 PMC2758742

[17] Grusche FA, Richardson HE, Harvey KF (2010) Upstream regulation of the hippo size control pathway. Curr Biol 20: R574–582. 20619814 10.1016/j.cub.2010.05.023

[18] Grzeschik NA, Parsons LM, Allott ML, Harvey KF, Richardson HE (2010) Lgl, aPKC, and Crumbs regulate the Salvador/Warts/Hippo pathway through two distinct mechanisms. Curr Biol 20: 573–581. 20362447 10.1016/j.cub.2010.01.055

[19] Yue T, Tian A, Jiang J (2012) The cell adhesion molecule echinoid functions as a tumor suppressor and upstream regulator of the Hippo signaling pathway. Dev Cell 22: 255–267. 22280890 10.1016/j.devcel.2011.12.011 PMC3288783

[20] Mao Y, Rauskolb C, Cho E, Hu WL, Hayter H, et al (2006) Dachs: an unconventional myosin that functions downstream of Fat to regulate growth, affinity and gene expression in Drosophila. Development 133: 2539–2551. 16735478 10.1242/dev.02427

[21] Rauskolb C, Pan G, Reddy BV, Oh H, Irvine KD (2011) Zyxin links fat signaling to the hippo pathway. PLoS Biol 9: e1000624. 21666802 10.1371/journal.pbio.1000624 PMC3110180

[22] Zhao M, Szafranski P, Hall CA, Goode S (2008) Basolateral junctions utilize warts signaling to control epithelial-mesenchymal transition and proliferation crucial for migration and invasion of Drosophila ovarian epithelial cells. Genetics 178: 1947–1971. 18430928 10.1534/genetics.108.086983 PMC2323789

[23] Skouloudaki K, Puetz M, Simons M, Courbard JR, Boehlke C, et al (2009) Scribble participates in Hippo signaling and is required for normal zebrafish pronephros development. Proc Natl Acad Sci U S A 106: 8579–8584. 19439659 10.1073/pnas.0811691106 PMC2688978

[24] Doggett K, Grusche FA, Richardson HE, Brumby AM (2011) Loss of the Drosophila cell polarity regulator Scribbled promotes epithelial tissue overgrowth and cooperation with oncogenic Ras-Raf through impaired Hippo pathway signaling. BMC Dev Biol 11: 57. 21955824 10.1186/1471-213X-11-57 PMC3206446

[25] Humbert P, Russell S, Richardson H (2003) Dlg, Scribble and Lgl in cell polarity, cell proliferation and cancer. Bioessays 25: 542–553. 12766944 10.1002/bies.10286

[26] Bilder D (2004) Epithelial polarity and proliferation control: links from the Drosophila neoplastic tumor suppressors. Genes Dev 18: 1909–1925. 15314019 10.1101/gad.1211604

[27] Humbert PO, Dow LE, Russell SM (2006) The Scribble and Par complexes in polarity and migration: friends or foes? Trends Cell Biol 16: 622–630. 17067797 10.1016/j.tcb.2006.10.005

[28] Lim J, Thiery JP (2011) Alternative path to EMT: regulation of apicobasal polarity in Drosophila. Dev Cell 21: 983–984. 22172667 10.1016/j.devcel.2011.11.017

[29] Woods DF, Hough C, Peel D, Callaini G, Bryant PJ (1996) Dlg protein is required for junction structure, cell polarity, and proliferation control in Drosophila epithelia. J Cell Biol 134: 1469–1482. 8830775 10.1083/jcb.134.6.1469 PMC2120992

[30] Woods DF, Bryant PJ (1991) The discs-large tumor suppressor gene of Drosophila encodes a guanylate kinase homolog localized at septate junctions. Cell 66: 451–464. 1651169 10.1016/0092-8674(81)90009-x

[31] Mechler BM, McGinnis W, Gehring WJ (1985) Molecular cloning of lethal(2)giant larvae, a recessive oncogene of Drosophila melanogaster. Embo J 4: 1551–1557. 3928370 10.1002/j.1460-2075.1985.tb03816.x PMC554381

[32] Bilder D, Li M, Perrimon N (2000) Cooperative regulation of cell polarity and growth by Drosophila tumor suppressors. Science 289: 113–116. 10884224 10.1126/science.289.5476.113

[33] Bilder D, Perrimon N (2000) Localization of apical epithelial determinants by the basolateral PDZ protein Scribble. Nature 403: 676–680. 10688207 10.1038/35001108

[34] Mathew D, Gramates LS, Packard M, Thomas U, Bilder D, et al (2002) Recruitment of scribble to the synaptic scaffolding complex requires GUK-holder, a novel DLG binding protein. Curr Biol 12: 531–539. 11937021 10.1016/s0960-9822(02)00758-3 PMC4661175

[35] Vaccari T, Bilder D (2009) At the crossroads of polarity, proliferation and apoptosis: the use of Drosophila to unravel the multifaceted role of endocytosis in tumor suppression. Mol Oncol 3: 354–365. 19560990 10.1016/j.molonc.2009.05.005 PMC2755045

[36] Rogulja D, Irvine KD (2005) Regulation of cell proliferation by a morphogen gradient. Cell 123: 449–461. 16269336 10.1016/j.cell.2005.08.030

[37] Badouel C, McNeill H (2009) Apical junctions and growth control in Drosophila. Biochim Biophys Acta 1788: 755–760. 18952051 10.1016/j.bbamem.2008.08.026

[38] Zeitler J, Hsu CP, Dionne H, Bilder D (2004) Domains controlling cell polarity and proliferation in the Drosophila tumor suppressor Scribble. J Cell Biol 167: 1137–1146. 15611336 10.1083/jcb.200407158 PMC2172630

[39] Hough CD, Woods DF, Park S, Bryant PJ (1997) Organizing a functional junctional complex requires specific domains of the Drosophila MAGUK Discs large. Genes Dev 11: 3242–3253. 9389655 10.1101/gad.11.23.3242 PMC316757

[40] Katoh Y, Katoh M (2006) Comparative integromics on FAT1, FAT2, FAT3 and FAT4. Int J Mol Med 18: 523–528. 16865240

[41] Viktorinova I, Konig T, Schlichting K, Dahmann C (2009) The cadherin Fat2 is required for planar cell polarity in the Drosophila ovary. Development 136: 4123–4132. 19906848 10.1242/dev.039099

[42] Dietzl G, Chen D, Schnorrer F, Su KC, Barinova Y, et al (2007) A genome-wide transgenic RNAi library for conditional gene inactivation in Drosophila. Nature 448: 151–156. 17625558 10.1038/nature05954

[43] Brumby AM, Richardson HE (2003) scribble mutants cooperate with oncogenic Ras or Notch to cause neoplastic overgrowth in Drosophila. Embo J 22: 5769–5779. 14592975 10.1093/emboj/cdg548 PMC275405

[44] Uhlirova M, Jasper H, Bohmann D (2005) Non-cell-autonomous induction of tissue overgrowth by JNK/Ras cooperation in a Drosophila tumor model. Proc Natl Acad Sci U S A 102: 13123–13128. 16150723 10.1073/pnas.0504170102 PMC1201591

[45] Chen CL, Schroeder MC, Kango-Singh M, Tao C, Halder G (2012) Tumor suppression by cell competition through regulation of the Hippo pathway. Proc Natl Acad Sci U S A 109: 484–489. 22190496 10.1073/pnas.1113882109 PMC3258595

[46] Igaki T, Pagliarini RA, Xu T (2006) Loss of cell polarity drives tumor growth and invasion through JNK activation in Drosophila. Curr Biol 16: 1139–1146. 16753569 10.1016/j.cub.2006.04.042

[47] Igaki T, Pastor-Pareja JC, Aonuma H, Miura M, Xu T (2009) Intrinsic tumor suppression and epithelial maintenance by endocytic activation of Eiger/TNF signaling in Drosophila. Dev Cell 16: 458–465. 19289090 10.1016/j.devcel.2009.01.002 PMC2729686

[48] Norman M, Wisniewska KA, Lawrenson K, Garcia-Miranda P, Tada M, et al (2012) Loss of Scribble causes cell competition in mammalian cells. J Cell Sci 125: 59–66. 22250205 10.1242/jcs.085803 PMC3269023

[49] Igaki T (2009) Correcting developmental errors by apoptosis: lessons from Drosophila JNK signaling. Apoptosis 14: 1021–1028. 19466550 10.1007/s10495-009-0361-7

[50] Bennett FC, Harvey KF (2006) Fat cadherin modulates organ size in Drosophila via the Salvador/Warts/Hippo signaling pathway. Curr Biol 16: 2101–2110. 17045801 10.1016/j.cub.2006.09.045

[51] Silva E, Tsatskis Y, Gardano L, Tapon N, McNeill H (2006) The tumor-suppressor gene fat controls tissue growth upstream of expanded in the hippo signaling pathway. Curr Biol 16: 2081–2089. 16996266 10.1016/j.cub.2006.09.004

[52] Willecke M, Hamaratoglu F, Kango-Singh M, Udan R, Chen CL, et al.. (2006) The Fat Cadherin Acts through the Hippo Tumor-Suppressor Pathway to Regulate Tissue Size. Curr Biol. 10.1016/j.cub.2006.09.005 16996265

[53] Cho E, Feng Y, Rauskolb C, Maitra S, Fehon R, et al (2006) Delineation of a Fat tumor suppressor pathway. Nat Genet 38: 1142–1150. 16980976 10.1038/ng1887

[54] Mao Y, Kucuk B, Irvine KD (2009) Drosophila lowfat, a novel modulator of Fat signaling. Development 136: 3223–3233. 19710173 10.1242/dev.036152 PMC2739141

[55] Cho E, Irvine KD (2004) Action of fat, four-jointed, dachsous and dachs in distal-to-proximal wing signaling. Development 131: 4489–4500. 15342474 10.1242/dev.01315

[56] Schroeder MC, Halder G (2012) Regulation of the Hippo pathway by cell architecture and mechanical signals. Semin Cell Dev Biol. 10.1016/j.semcdb.2012.06.001 22750148

[57] Robinson BS, Huang J, Hong Y, Moberg KH (2010) Crumbs regulates Salvador/Warts/Hippo signaling in Drosophila via the FERM-domain protein Expanded. Curr Biol 20: 582–590. 20362445 10.1016/j.cub.2010.03.019 PMC2855393

[58] Grzeschik NA, Amin N, Secombe J, Brumby AM, Richardson HE (2007) Abnormalities in cell proliferation and apico-basal cell polarity are separable in Drosophila lgl mutant clones in the developing eye. Dev Biol 311: 106–123. 17870065 10.1016/j.ydbio.2007.08.025 PMC2974846

[59] Leong GR, Goulding KR, Amin N, Richardson HE, Brumby AM (2009) Scribble mutants promote aPKC and JNK-dependent epithelial neoplasia independently of Crumbs. BMC Biol 7: 62. 19778415 10.1186/1741-7007-7-62 PMC2760524

[60] Grusche FA, Degoutin JL, Richardson HE, Harvey KF (2011) The Salvador/Warts/Hippo pathway controls regenerative tissue growth in Drosophila melanogaster. Dev Biol 350: 255–266. 21111727 10.1016/j.ydbio.2010.11.020

[61] Hafezi Y, Bosch JA, Hariharan IK (2012) Differences in levels of the transmembrane protein Crumbs can influence cell survival at clonal boundaries. Dev Biol 368: 358–369. 22683826 10.1016/j.ydbio.2012.06.001 PMC3412113

[62] Ling C, Zheng Y, Yin F, Yu J, Huang J, et al (2010) The apical transmembrane protein Crumbs functions as a tumor suppressor that regulates Hippo signaling by binding to Expanded. Proc Natl Acad Sci U S A 107: 10532–10537. 20498073 10.1073/pnas.1004279107 PMC2890787

[63] Chen CL, Gajewski KM, Hamaratoglu F, Bossuyt W, Sansores-Garcia L, et al (2010) The apical-basal cell polarity determinant Crumbs regulates Hippo signaling in Drosophila. Proc Natl Acad Sci U S A 107: 15810–15815. 20798049 10.1073/pnas.1004060107 PMC2936591

[64] Grzeschik NA, Parsons LM, Richardson HE (2010) Lgl, the SWH pathway and tumorigenesis: It’s a matter of context & competition! Cell Cycle. 9: 3202–3212. 10.4161/cc.9.16.12633 20724829

[65] Froldi F, Ziosi M, Garoia F, Pession A, Grzeschik NA, et al (2010) The lethal giant larvae tumour suppressor mutation requires dMyc oncoprotein to promote clonal malignancy. BMC Biol 8: 33. 20374622 10.1186/1741-7007-8-33 PMC2877678

[66] Menendez J, Perez-Garijo A, Calleja M, Morata G (2010) A tumor-suppressing mechanism in Drosophila involving cell competition and the Hippo pathway. Proc Natl Acad Sci U S A 107: 14651–14656. 20679206 10.1073/pnas.1009376107 PMC2930466

[67] Sun G, Irvine KD (2011) Regulation of Hippo signaling by Jun kinase signaling during compensatory cell proliferation and regeneration, and in neoplastic tumors. Dev Biol 350: 139–151. 21145886 10.1016/j.ydbio.2010.11.036 PMC3038240

[68] Zhao B, Lei QY, Guan KL (2008) The Hippo-YAP pathway: new connections between regulation of organ size and cancer. Curr Opin Cell Biol. 10.1016/j.ceb.2008.10.001 PMC3296452 18955139

[69] Zhao B, Li L, Lei Q, Guan KL (2010) The Hippo-YAP pathway in organ size control and tumorigenesis: an updated version. Genes Dev 24: 862–874. 20439427 10.1101/gad.1909210 PMC2861185

[70] Halder G, Dupont S, Piccolo S (2012) Transduction of mechanical and cytoskeletal cues by YAP and TAZ. Nat Rev Mol Cell Biol. 10.1038/nrm3416 22895435

[71] Badouel C, Gardano L, Amin N, Garg A, Rosenfeld R, et al (2009) The FERM-domain protein Expanded regulates Hippo pathway activity via direct interactions with the transcriptional activator Yorkie. Dev Cell 16: 411–420. 19289086 10.1016/j.devcel.2009.01.010

[72] Oh H, Irvine KD (2008) In vivo regulation of Yorkie phosphorylation and localization. Development 135: 1081–1088. 18256197 10.1242/dev.015255 PMC2387210

[73] Oh H, Irvine KD (2009) In vivo analysis of Yorkie phosphorylation sites. Oncogene 28: 1916–1927. 19330023 10.1038/onc.2009.43 PMC2701235

[74] Oh H, Reddy BV, Irvine KD (2009) Phosphorylation-independent repression of Yorkie in Fat-Hippo signaling. Dev Biol. 10.1016/j.ydbio.2009.08.026 PMC2774787 19733165

[75] Dong J, Feldmann G, Huang J, Wu S, Zhang N, et al (2007) Elucidation of a universal size-control mechanism in Drosophila and mammals. Cell 130: 1120–1133. 17889654 10.1016/j.cell.2007.07.019 PMC2666353

[76] Sopko R, Silva E, Clayton L, Gardano L, Barrios-Rodiles M, et al (2009) Phosphorylation of the tumor suppressor fat is regulated by its ligand Dachsous and the kinase discs overgrown. Curr Biol 19: 1112–1117. 19540118 10.1016/j.cub.2009.05.049 PMC2851237

[77] Tyler DM, Baker NE (2007) Expanded and fat regulate growth and differentiation in the Drosophila eye through multiple signaling pathways. Dev Biol 305: 187–201. 17359963 10.1016/j.ydbio.2007.02.004 PMC2075468

[78] Feng Y, Irvine KD (2007) Fat and expanded act in parallel to regulate growth through warts. Proc Natl Acad Sci U S A 104: 20362–20367. 18077345 10.1073/pnas.0706722105 PMC2154436

